# Temporally Validated and Laboratory-Reconciled Soft Sensing of Xylene-Soluble Fraction in Industrial Polypropylene Manufacturing

**DOI:** 10.3390/polym18192401

**Published:** 2026-10-01

**Authors:** Joaquín Hernández-Fernández

**Affiliations:** 1 Chemistry Program, Department of Natural and Exact Sciences, University of Cartagena, San Pablo Campus, Cartagena de Indias 130015, Colombia; jhernandezf@unicartagena.edu.co; 2 Department of Natural and Exact Science, Universidad de la Costa, Barranquilla 080002, Colombia; 3 Institute of Materials Technology (ITM), Universitat Politecnica de Valencia (UPV), Plaza Ferrandiz and Carbonell s/n, 03801 Alcoy, Spain; 4 Grupo de Investigación GIA, Fundacion Universitaria Tecnologico Comfenalco, Cr 44 D N 30A, 91, Cartagena 130015, Colombia

**Keywords:** polypropylene, xylene-soluble fraction, soft sensor, machine learning, predictive modeling, temporal validation, process monitoring

## Abstract

The xylene-soluble fraction (XS) is a critical quality attribute in polypropylene manufacturing because it is associated with polymer architecture, catalyst stereoselectivity, comonomer incorporation, and polymerization conditions. Its conventional determination by ASTM D5492 is laboratory-based and cannot provide continuous information on the evolving quality state of an industrial reactor. This study addresses how much XS predictability originates from the previously known analytical state and how much is attributable to the contemporaneous reactor state. Of the 425 original industrial records included, one physically invalid feed condition was excluded, leaving 425 curated observations. These observations were evaluated chronologically rather than via random row-wise partitioning, and product code and downstream quality descriptors that were unavailable at prediction time were excluded from the primary formulation. The process-only model achieved a final temporal-test R^2^ of 0.666, whereas historical analytical/recipe information alone yielded R^2^ = 0.843. Integration of both information sources increased performance to R^2^ = 0.902, RMSE = 0.640 wt.%, and MAE = 0.306 wt.%. Expanding-window temporal validation yielded a mean R^2^ of 0.875 ± 0.115. The strongest independently supported domain was Family H, for which 50 future observations were predicted with R^2^ = 0.930, RMSE = 0.304 wt.%, and MAE = 0.188 wt.%. The results support a laboratory-reconciled soft-sensor architecture in which ASTM D5492 serves as the certification reference, while the model provides intermediate estimates between laboratory measurements.

## 1. Introduction

Polypropylene (PP) is one of the largest-volume polymers worldwide, accounting for a substantial fraction of global non fiber plastic production, with applications in packaging, films, fibers, automotive components, and impact-resistant copolymers [1]. Its industrial production relies predominantly on Ziegler–Natta and metallocene catalytic systems operated under different polymerization configurations [2]. Among the relevant microstructural quality descriptors, the xylene soluble fraction (XS) is particularly important because it is associated with the amorphous or low-crystallinity fraction of PP and is sensitive to changes in polymer architecture, stereoregularity, comonomer incorporation, and catalyst-dependent microstructure [2]. Because XS is not directly measurable in the reactor, its industrial determination relies on offline laboratory analysis. ASTM D5492 is a standardized reference method for determining the xylene-soluble fraction of propylene plastics [3]. Although analytically established, conventional gravimetric determination requires relatively large quantities of polymer and solvent and involves long extraction times [4]. Alternative laboratory approaches have therefore been investigated. Boyron et al. proposed a size exclusion chromatography (SEC)-based method that substantially reduces analysis time while producing estimates comparable with standardized extraction methods [4]. Shi et al. investigated the relationship between conventional xylene-soluble measurements and crystallization elution fractionation (CEF) [5], whereas Melo et al. examined the liquid–liquid equilibrium underlying the XS separation procedure from a thermodynamic perspective [6].

Soft sensors provide a complementary strategy for bridging the temporal gap between reactor operation and laboratory quality measurements by estimating difficult-to-measure variables from routinely available process information. Their design and application in polymer processing have been extensively reviewed by Abeykoon [7]. In polymerization processes, different data driven architectures have been investigated, including deep belief networks for melt index prediction [8], long short term memory networks for capturing temporal process behavior [9], recursive partial least squares models for grade change operations [10], combined local Gaussian process regression for multimode polypropylene production [11], weighted least squares support vector machines [12], and clustering-based hybrid soft sensors for polypropylene grade change operation [13]. These studies demonstrate that the melt index has historically received substantial attention as a target quality variable. Direct virtual sensing of XS in industrial polypropylene has also been reported [14], although this application remains considerably less explored than melt index prediction.

More recent studies have expanded machine assisted quality prediction in polyolefin manufacturing. Machine-learning approaches have been applied to process structure property relationships in polypropylene [15], multivariate industrial soft sensing for polyethylene quality variables [16], dynamic graph neural network prediction of polypr pylene melt index [17], and graph based soft sensor architectures incorporating feature expansion and multi-hop attention [18]. In parallel, explainability and transparency have become increasingly important in chemical process engineering because predictive accuracy alone does not establish physicochemical causality or guarantee trustworthy industrial interpretation [19,20]. Feature attribution approaches such as SHAP, which are grounded in Shapley value concepts, provide a systematic framework for interpreting model predictions [21], although such attributions should be interpreted as model dependent statistical explanations rather than direct evidence of causal chemical mechanisms.

## 2. Materials and Methods

### 2.1. Industrial Polypropylene Manufacturing Process

**Figure 1:**
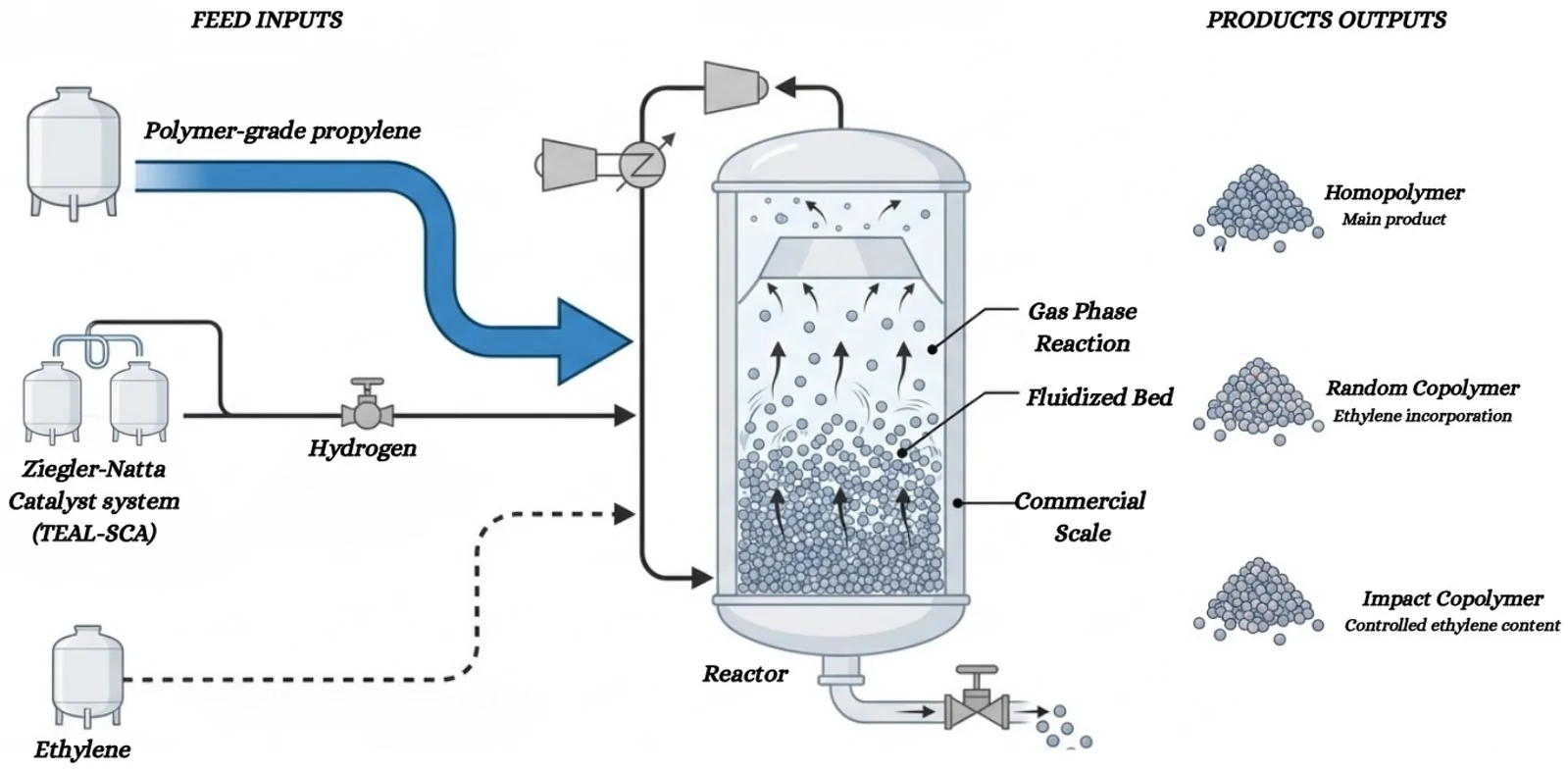
Representation of the industrial UNIPOL gas-phase polypropylene manufacturing process.

The polypropylene samples evaluated in this study were produced in a large-scale industrial unit based on UNIPOL polypropylene process technology. Polypropylene samples were produced in a commercial UNIPOL gas-phase fluidized-bed reactor (W. R. Grace & Co., Columbia, MD, USA) using polymer-grade propylene, a Ziegler–Natta catalyst system (TEAL/SCA), hydrogen as a molecular-weight regulator, and ethylene when copolymer grades were required. The industrial operation covered different polypropylene families, including homopolymer, random copolymer, and copolymer grades. Homopolymer grades were produced primarily from propylene, whereas random and copolymer grades involved controlled incorporation of ethylene. To preserve industrial confidentiality, proprietary catalyst formulations, exact reactor geometry, plant capacity, and grade identities are not disclosed. See Figure 1.

### 2.2. Industrial Dataset and Temporal Structure

The original database contained 425 industrial production records from routine plant and quality-control systems. One record containing a physically invalid combination of feed conditions was excluded during data-quality auditing, leaving 425 curated observations (see Table S1). Records were ordered chronologically according to production time. Predictor eligibility was determined from physical meaning and availability at the intended prediction time. Product code, current XS, melt-flow index (MF), contemporaneous laboratory ethylene content (Et), and other downstream quality descriptors were excluded from the primary soft-sensor formulation because they either encode product specifications or are unavailable when prediction is required (see Table S2).

The predictors listed in Table 1 were organized into three predefined information scenarios according to their availability at the intended prediction time. Scenario I (current reactor state) included exclusively contemporaneous process variables available at time t. Scenario II (historical analytical/recipe state) included only information available before time *t*, including the most recent certified ASTM D5492 XS result, lagged XS descriptors, rolling historical XS descriptors, and historical recipe information. Scenario III (laboratory-reconciled state) combined the complete predictor sets from Scenarios I and II without introducing product identity or downstream quality variables. All three scenarios were evaluated using the same chronological data partitions and preprocessing workflow summarized in Table 2, ensuring that differences in predictive performance reflected differences in information content rather than differences in validation design.

### 2.3. Laboratory Determination of Xylene-Soluble Fraction

**Figure 2:**
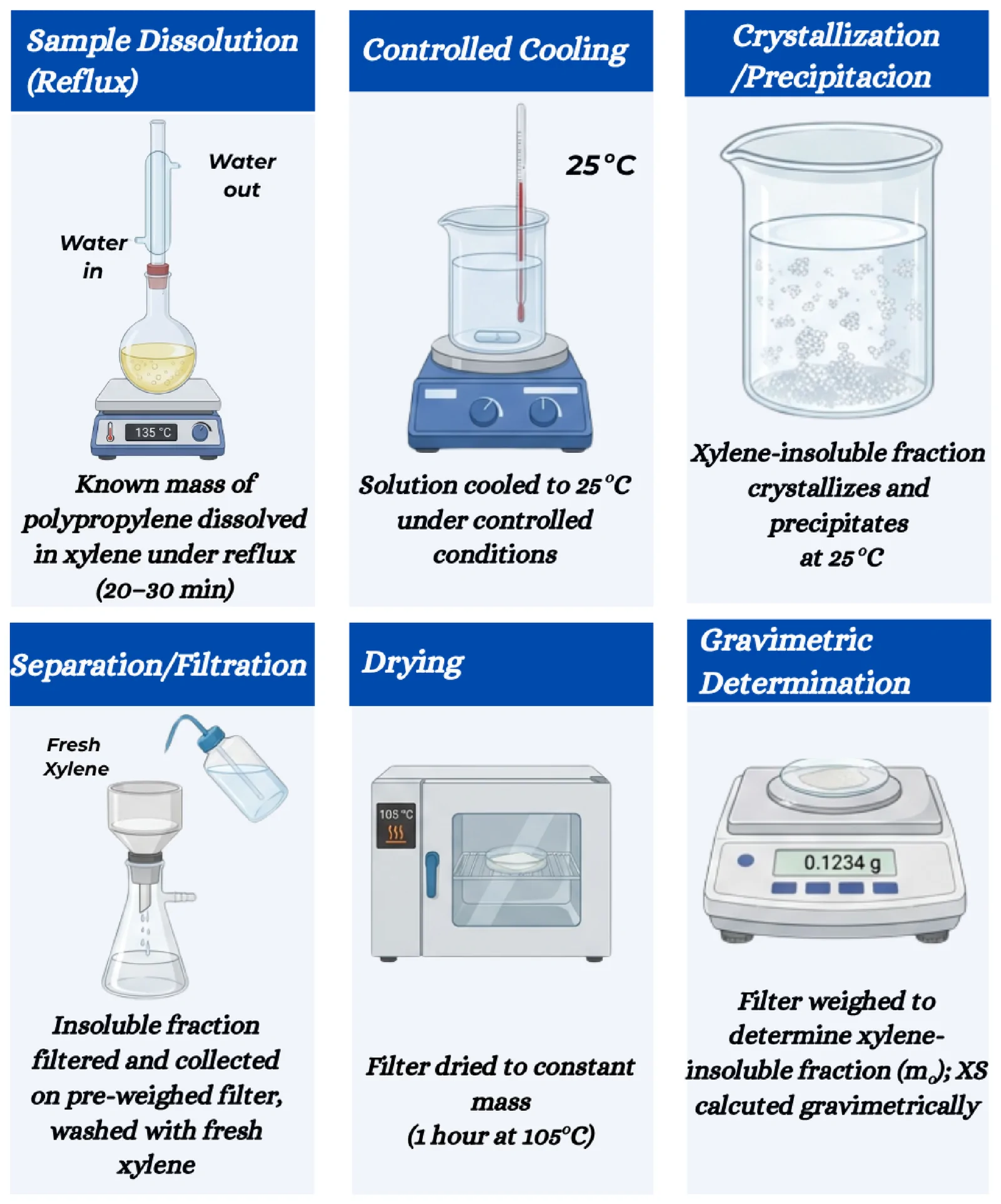
Determination of xylene-soluble fraction according to ASTM D5492.

The xylene-soluble fraction was determined according to ASTM D5492, “Standard Test Method for Determination of Xylene Solubles in Propylene Plastics.” This method is intended for the determination of the xylene-soluble fraction at 25 °C in polypropylene homopolymers and copolymers.See Figure 2. The XS value is expressed as the mass percentage of polymer remaining soluble under the method’s standardized conditions. The xylene-soluble fraction was calculated using the following expression:

$$\mathrm{XS}\;(\%)\;=\;\frac{m_{s}}{m_{0}}\;\times 100$$

where m_s_ is the mass of the xylene-soluble fraction, and m_0_ is the initial polymer mass. All XS values used in this study correspond to certified laboratory measurements generated as part of the routine industrial quality control program. Therefore, the response variable reflects actual commercial quality-control data rather than laboratory-scale experimental measurements. Since the soluble fraction is commonly associated with the amorphous or low-crystallinity fraction of polypropylene, XS was used as a critical quality attribute for evaluating polymer microstructure and process stability.

### 2.4. Data-Quality, Missingness, and Algebraic-Dependency Audit

Before model development, the database was audited for missing values, non-numeric codes, physically implausible entries, structured missingness, and algebraic redundancy. One observation with negative TEAL flow, along with zero catalyst and SCA feeds, was excluded as physically invalid. Missingness was not assumed to be random because several descriptors are production-mode-specific; C_2_/C_3_, for example, was recorded in 74 of the 425 curated observations. Missing-value handling was learned from development data only. Numerical missing values were imputed using development-set medians, and categorical missing values, when present, were represented by an explicit missing level. Categorical variables were retained in a candidate model were one-hot encoded using categories learned exclusively from development data; unseen validation/test levels were ignored rather than refitting the encoder. Standardization was fitted to the development data and used only for PCA and scale sensitive linear models; tree ensembles were fitted to the unscaled processed matrix. The revised workflow used only information available before or at the time of prediction. Historical analytical descriptors were generated causally from ASTM D5492 results preceding the current observation, including the most recently available XS value and rolling summaries of earlier XS measurements within the relevant historical production context. No contemporaneous or future XS value was used to construct a predictor. Product code, current XS, MF, contemporaneous laboratory Et, and other downstream quality control results were excluded from the primary online predictor set. See Table S3.

### 2.5. Statistical and Architecture-Aware Analysis

Descriptive statistics characterized XS and the operating space. Differences among the H, C, and R polypropylene families were evaluated using one-way ANOVA and the Kruskal–Wallis test, followed by Tukey HSD pairwise comparisons. Pearson, Spearman, and Kendall coefficients were used for descriptive purposes. Because pooled correlations can combine within-regime process sensitivity with between-family differences, no pooled coefficient was interpreted as direct causal evidence.

### 2.6. Multivariate Analysis

Principal component analysis (PCA) was applied as an exploratory representation of the standardized industrial operating space. Scores and loadings were used to visualize multimodality, collinearity, and family-dependent operating domains. PCA was not used to infer causal mechanisms or as a substitute for temporal validation.

### 2.7. Temporal Machine-Learning Development and Validation

The 425 curated observations were chronologically divided into development/training (*n* = 296), temporal validation (*n* = 63), and final future test (*n* = 65) period(s See Table S4). No random row-wise split was used. Preprocessing, imputation, encoding, feature construction, model selection, and hyperparameter optimization were restricted to the development and validation sets. Candidate models included regularized/latent-variable regression and tree ensembles. Selection considered validation R^2^, RMSE, MAE, temporal stability, residual behavior, and parsimony. The final model included Extra Trees with 300 estimators, max_features_ = 0.5, min_samplesleaf_ = 2, unrestricted depth, and random Max_state_ = 42. After the workflow was frozen, the 65-record test period was evaluated once. R^2^, RMSE, and MAE for each comparison were calculated from the same observed/predicted vector.

Expanding-window validation preserved the original chronological ordering of the observations. For each fold, the training interval contained all observations available before the corresponding validation interval, and the training window was progressively enlarged without reintroducing later observations into earlier folds. The complete fold definitions, including chronological record ranges and sample sizes, are reported in Table S5. The final 65-observation temporal test set remained completely isolated from this procedure and was evaluated only after preprocessing, feature construction, model selection, and hyperparameter settings had been frozen [22].

### 2.8. Information Decomposition and Model Interpretation

Three predefined information scenarios were evaluated using the predictor sets specified in Table 1. Scenario I (“current reactor state”) contained exclusively contemporaneous process measurements available at prediction time. Scenario II (“historical analytical/recipe state”) contained exclusively causal historical descriptors constructed from information available before the target observation. Scenario III (“laboratory-reconciled state”) combined Scenarios I and II without introducing any additional contemporaneous or downstream quality variables. The same chronological partitions, preprocessing rules, model architecture, and evaluation metrics were used across the three scenarios so that differences in predictive performance reflected differences in information content rather than differences in validation design.

### 2.9. Laboratory-Reconciled Industrial Soft Sensor

**Figure 3:**
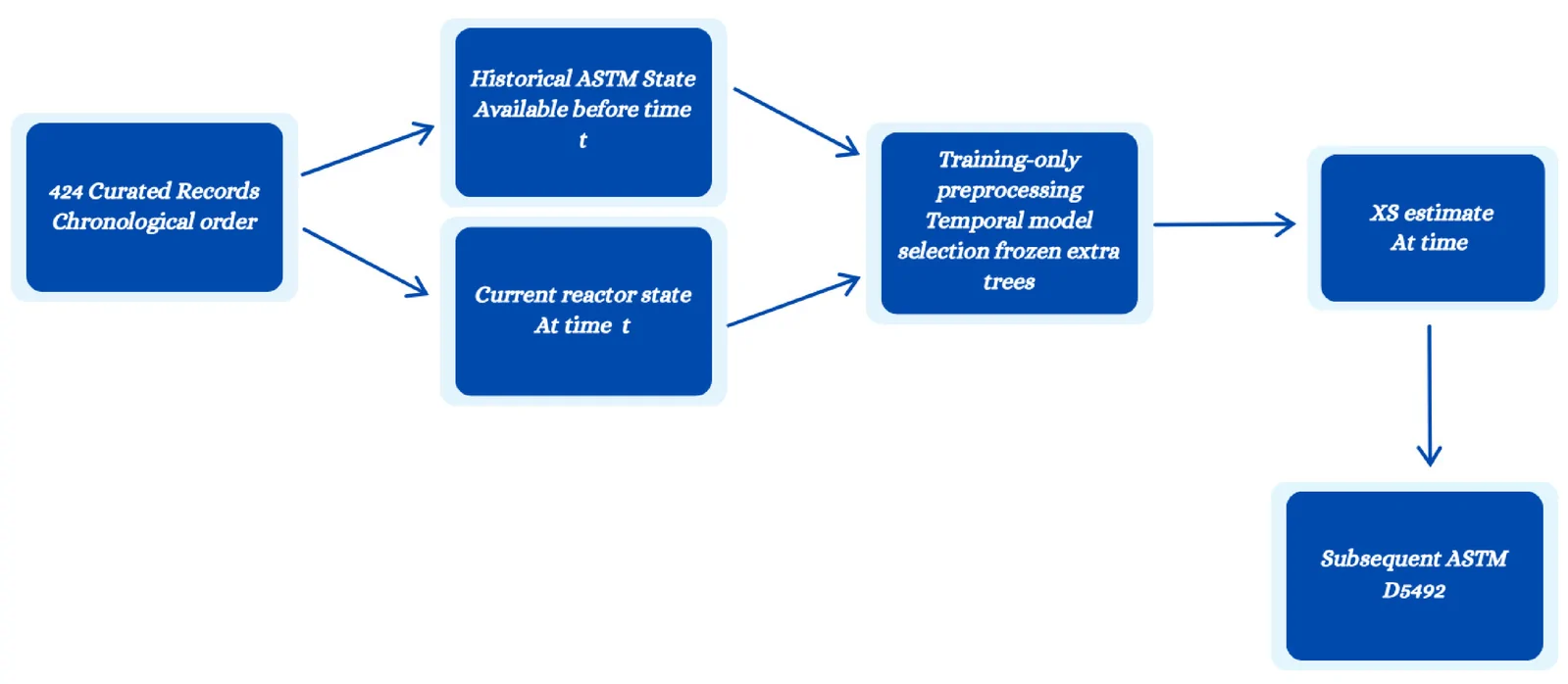
Leakage-controlled temporal information flow. Historical ASTM information and the current reactor state are processed using transformations fitted on development data only; the final future test period is not used for model selection.

The final architecture was formulated as a sequential laboratory-reconciled soft sensor. At prediction time *t*, the estimator receives only historical ASTM D5492 information available before t together with current reactor-state variables. When the subsequent certified ASTM result becomes available, it may be incorporated only into future predictions. ASTM D5492 therefore remains the certification reference, while the model provides intermediate estimates for process monitoring and early detection of deviations. See Figure 3.

## 3. Results and Discussion

### 3.1. Industrial Heterogeneity and Statistical Structure of XS

**Figure 4:**
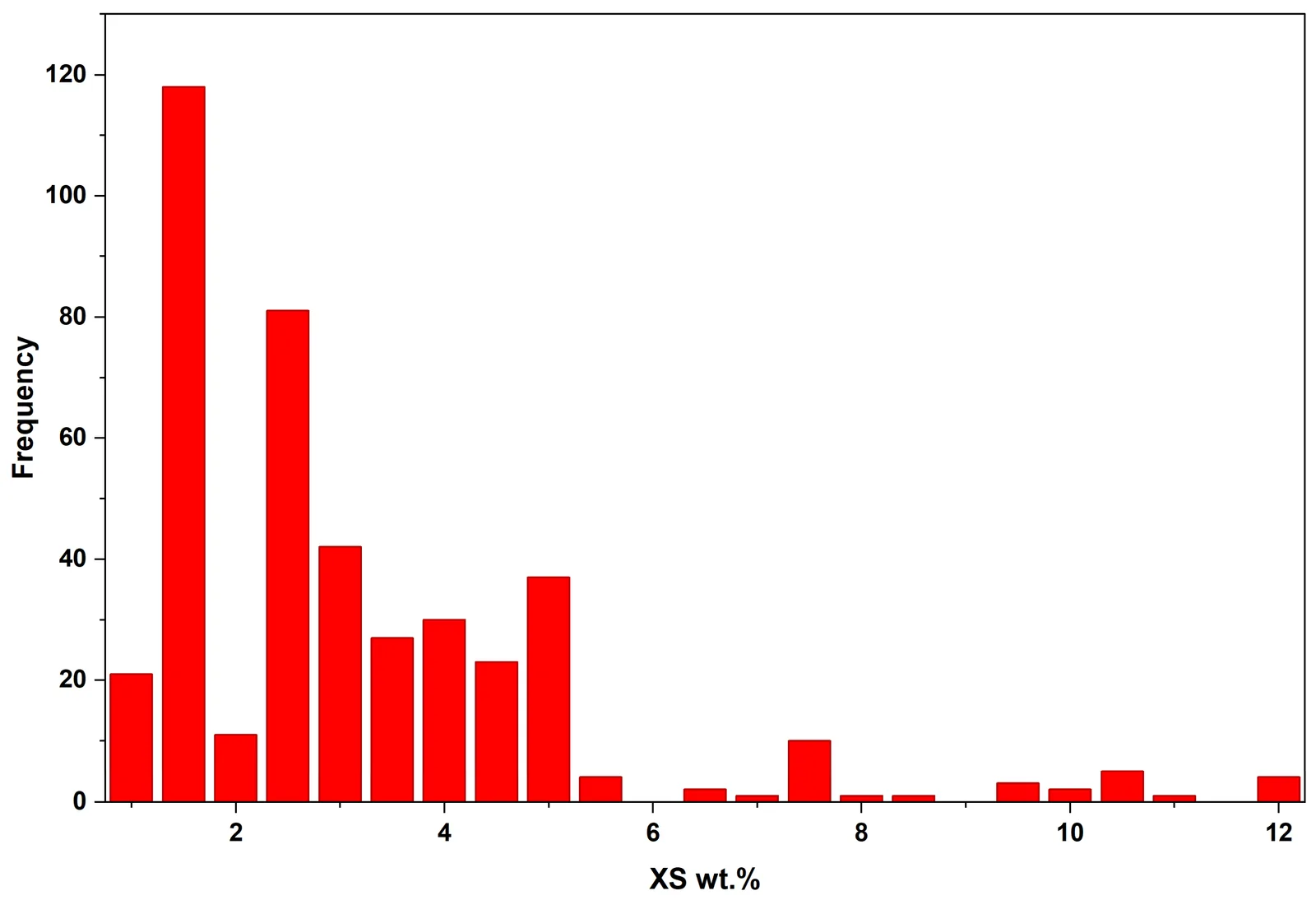
Distribution of XS in the 425-record curated industrial dataset.

The 425 record curated dataset spans an XS interval of 0.979–11.780 wt.% with a mean of 3.155 wt.% and SD of 2.085 wt.% (CV = 66.08%). The large coefficient of variation and the right-skewed response (skewness = 1.898; kurtosis = 4.267) show that a single narrow Gaussian response distribution does not well represent the industrial portfolio. See Table 3. More importantly, this dispersion is chemically structured rather than being attributable only to random process noise: the database combines polypropylene families produced under different catalyst/donor, hydrogen, and comonomer regimes. Consequently, the statistical problem is intrinsically multimodal, and the total XS variance contains both between-regime and within regime components. This distinction is essential because a predictor can appear highly informative in pooled data merely by discriminating regimes with different mean XS values, without explaining fluctuations within a given production domain. See Figure 4.

The process descriptors show the same heterogeneity. Ethylene related variables are structurally sparse because ethylene is not required in every production regime, whereas gas phase and catalyst feed variables are broadly available. Several descriptors are also strongly skewed or leptokurtic, including H_2_ Feed, [C_2_H_4_], and [H_2_]. These distributions were therefore treated as properties of an industrial multigrade process rather than as automatic grounds for deleting extreme observations. The revised workflow instead distinguishes physically invalid records from valid low frequency operating states and prevents the latter from being removed solely because they are statistically unusual. This is important for a quality soft sensor because rare operating states may be precisely those associated with grade transitions or increased off specification risk.

From a physicochemical perspective, XS is an integrated response rather than a directly manipulated reactor variable. The measured soluble fraction depends on the population of chains that fails to crystallize under the standardized ASTM D5492 separation conditions. That population is influenced jointly by stereoregularity, molar mass distribution, comonomer incorporation, and the distribution of catalytic sites. Accordingly, the broad XS distribution should not be interpreted as evidence of a single dominant control variable. It is more appropriately viewed as the observable outcome of several coupled kinetic and structural processes whose relative importance changes with production regime.

This interpretation also establishes an important limit on inference. ASTM D5492 provides a relative measure of the soluble fraction; it does not independently quantify tacticity, crystallinity, molecular-weight distribution, or comonomer sequence distribution. Therefore, changes in XS are discussed here as being compatible with changes in polymer microstructure rather than as direct reconstructions of a unique microstructural state.

### 3.2. Polymer-Family Heterogeneity and Between-Domain Variance

**Figure 5:**
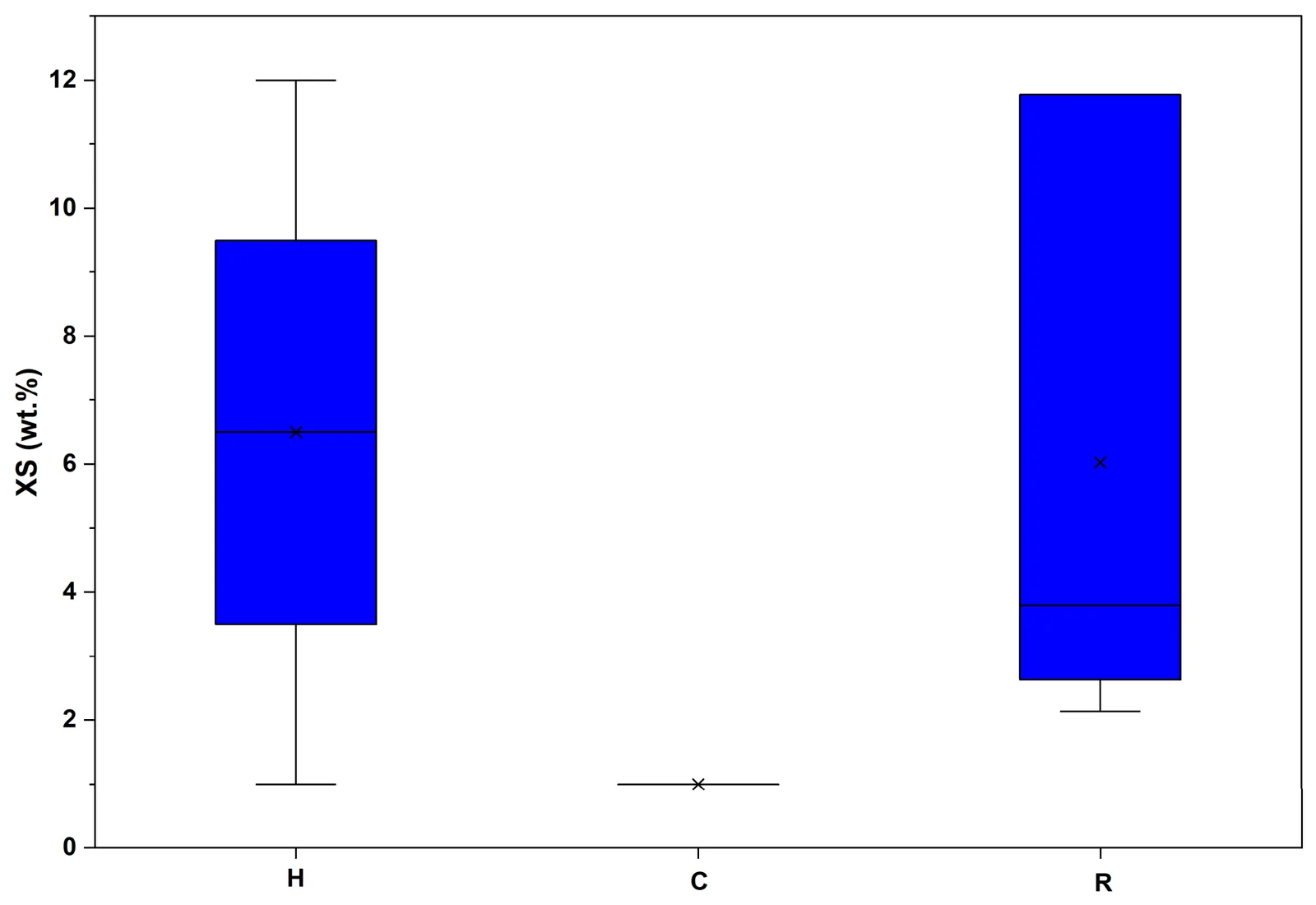
Distribution of XS by polypropylene family in the curated industrial dataset. The *y*-axis is on the same scale as the values summarized in Table 4. The horizontal line within each box represents the median XS value, the blue box represents the interquartile range between the first and third quartiles, and the cross symbol (×) indicates the mean XS value.

After exclusion of the physically invalid record, the family-resolved analysis was performed using the same 425 observation curated dataset employed throughout the study. As summarized in Table 4, the three polypropylene families exhibit clearly differentiated XS distributions. Family C occupies the lowest and narrowest XS domain, Family H shows an intermediate and substantially broader distribution, and Family R exhibits the highest XS levels. These differences are statistically significant according to both one-way ANOVA and the Kruskal–Wallis test, while Tukey HSD confirms significant pairwise differences among the three families (all adjusted *p* < 0.001). See Table 5. The agreement between parametric and rank-based analyses indicates that the observed family dependence is robust and is not an artifact of assuming normality of the XS distribution. See Figure 5.

The magnitude of the pairwise differences is also chemically meaningful. The mean separation between C and H is 1.9009 wt.%, between H and R is 3.8859 wt.%, and between C and R is 5.7868 wt.%. These differences are much larger than the within-family SD of C and remain substantial relative to the SDs of H and R. See Table 6. The family effect, therefore, represents a major between domain contribution to the total XS variance. In mechanistic terms, this is consistent with changes in polymer architecture and comonomer content altering the population of crystallizable sequences. However, because family is also associated with recipe and operating window, these statistics do not identify which individual process variable causes the separation. For this reason, family is treated as an operating domain descriptor, and family resolved predictive performance is reported explicitly rather than allowing a global model to obtain apparently high accuracy by learning only between-family differences.

The pooled correlation matrix should therefore be interpreted conditionally. If Y denotes XS, X is a process variable, and G is the production regime, and a pooled association Corr(X, Y) contains both within-regime covariance and covariance induced by differences in E[X|G] and E[Y|G]. Consequently, a large pooled coefficient can arise even when the within-regime relationship is weak, and a weak pooled coefficient can mask relationships with opposite or nonlinear behavior across regimes. This provides a statistical explanation for the contradictions in the earlier manuscript, in which hydrogen and ethylene were assigned different signs or strengths depending on the analysis. The revised manuscript does not rely on a single pooled coefficient as evidence for a universal chemical mechanism.

Family dependence is therefore treated as evidence of operating-domain heterogeneity. Family-resolved performance is reported explicitly so that a strong global metric cannot conceal weak generalization within under-represented domains.

### 3.3. Operating-Space Confounding and Physicochemical Interpretation of Pooled Associations

**Figure 6:**
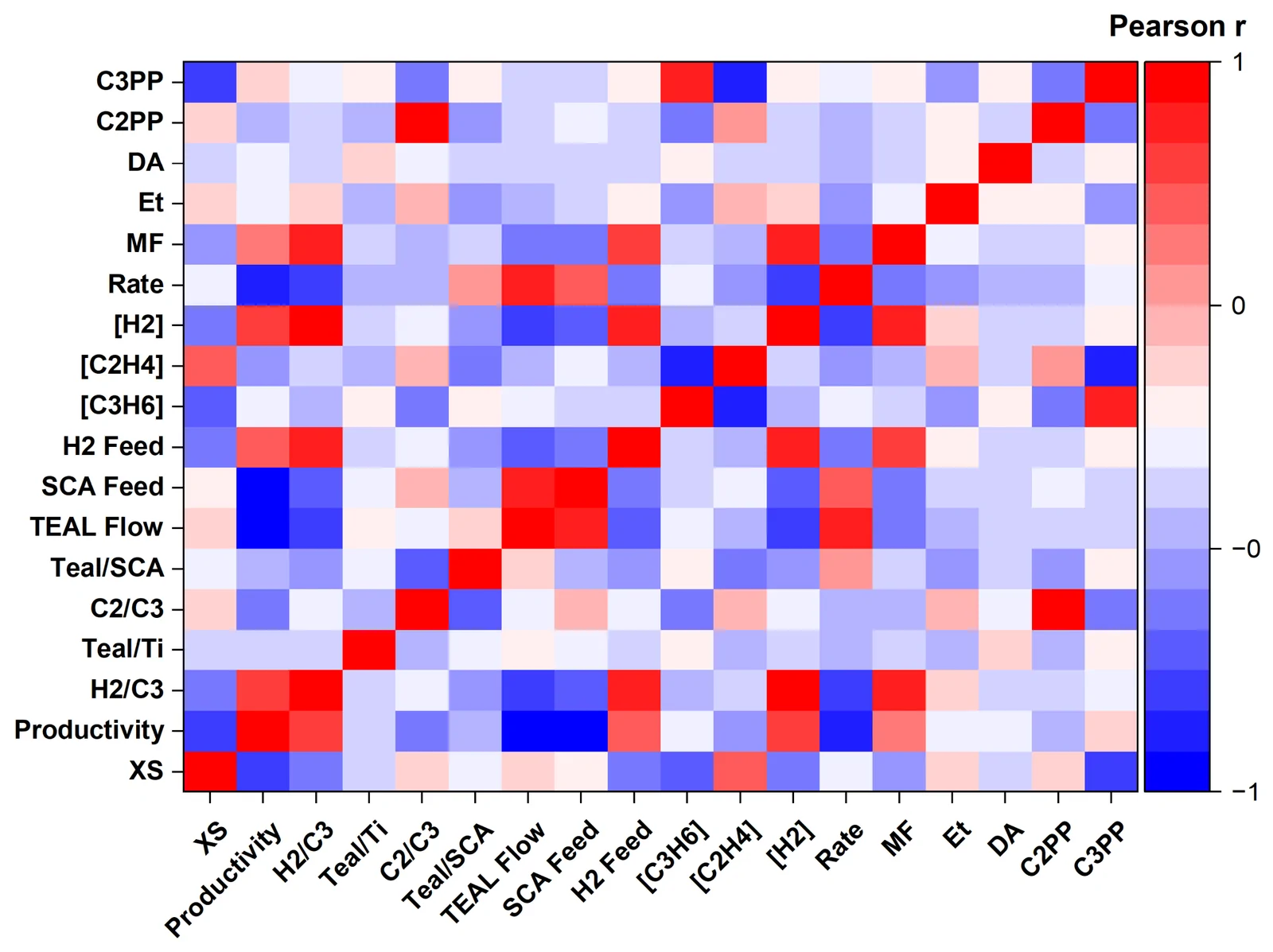
Pearson correlation heatmap.

The pooled correlation matrix in Figure 6 is retained for exploratory purposes. Correlations between XS and hydrogen-, comonomer-, or catalyst-related descriptors combine within-regime process sensitivity with differences between polymer families and recipes. Consequently, the absence of a pooled correlation should not be interpreted as a universal mechanistic effect. Hydrogen remains mechanistically relevant as a chain-transfer agent, ethylene as a comonomer affecting chain regularity, and donor/catalyst chemistry as a determinant of stereoselectivity. Still, the statistical magnitude of these associations is domain-dependent.

The chemical interpretation is instead anchored in established reaction pathways. Propagation at a Ziegler–Natta active site may be represented schematically as P_n* + CH_2_ = CH-CH_3_ → P_(n + 1)*_, whereas hydrogen introduces a competing chain-transfer pathway, P _n*_ + H_2_ → P_n-H_ + regenerated catalytic species. Increasing hydrogen, therefore, changes chain-length statistics and molecular-weight distribution, but the resulting effect on XS depends on stereospecific site populations, donor chemistry, residence history, and polymer architecture. Similarly, insertion of ethylene, P_n*_ + CH_2_ = CH_2_ → P_n_-CH_2_-CH_2_*, interrupts long propylene sequences and can reduce crystal perfection. These mechanisms justify considering hydrogen-, donor-, and ethylene-related variables as chemically plausible predictors; they do not imply that their pooled correlations are causal coefficients.

### 3.4. Multivariate Operating-Space Structure

**Figure 7:**
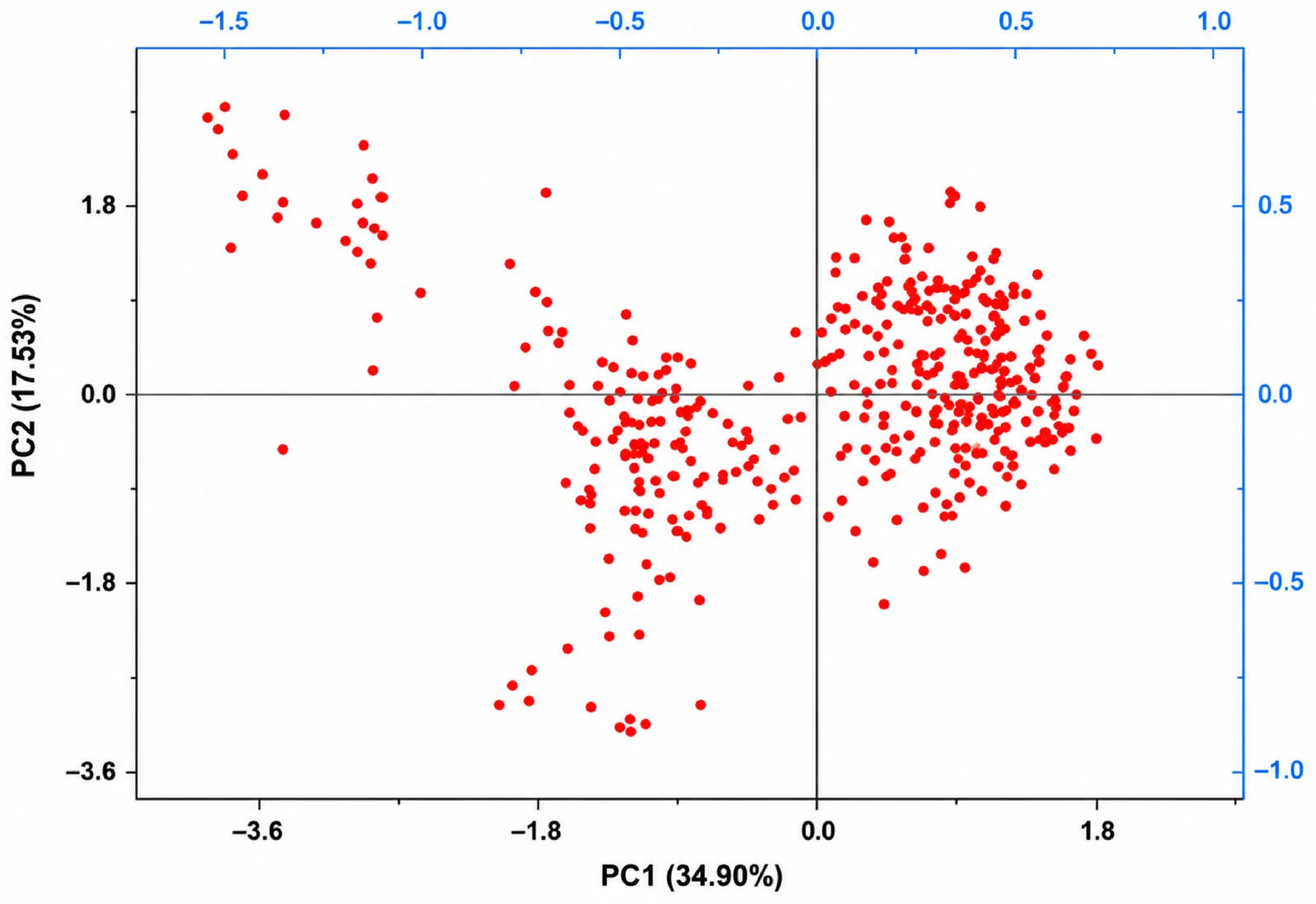
PCA scores and loadings generated from the same standardized process-only matrix and the same PCA fit. PC1 explains 34.90% and PC2 17.53% of the variance in both panels.

PCA provides an independent view of the same statistical structure. In the revised single fit PCA, PC1 explains 34.90% and PC2 17.53% of the standardized process variance for a cumulative 52.43% in the first two components. See Figure 7. Thus, approximately 47.57% of process variance remains outside the two dimensional projection, which is why the score map is used only to visualize dominant operating structure rather than to claim complete separation of production mechanisms. The fact that scores and loadings now originate from the same fit also makes the geometric interpretation internally consistent.

### 3.5. Quantitative Decomposition of Historical and Dynamic Predictive Information

The most important quantitative result is the decomposition of predictive information. With the same 65-record future test period, current reactor information alone yields R^2^ = 0.666, RMSE = 1.184 wt.%, and MAE = 0.533 wt.%. Thus, instantaneous process information contains substantial signal but leaves 33.4% of the test-set variance unexplained. Historical analytical/recipe information alone increases R^2^ to 0.843 and reduces RMSE to 0.812 wt.% and MAE to 0.416 wt.%. The improvement from process only to historical state prediction indicates that XS possesses substantial temporal and regime persistence: the recent analytical state is more informative about the next laboratory result than a static snapshot of the reactor considered in isolation.

The combined laboratory reconciled model reaches R^2^ = 0.902, RMSE = 0.640 wt.%, and MAE = 0.306 wt.%. The increase in R^2^ from 0.843 to 0.902 corresponds to an absolute gain of 0.059, but the more informative quantity is the reduction in residual variance. The unexplained fraction decreases from 1 − 0.843 = 0.157 to 1 − 0.902 = 0.098; therefore, incorporation of the contemporaneous reactor state removes approximately (0.157 − 0.098)/0.157 = 37.6% of the variance that remained unexplained after accounting for historical state. In parallel, RMSE decreases by 21.2% and MAE by 26.4%. These three metrics converge on the same conclusion: current reactor measurements provide genuine incremental predictive information rather than merely duplicating the historical ASTM signal. See Table 7.

### 3.6. Temporal Generalization, Concept Drift, and Domain-Resolved Performance

Temporal validation is equally important because industrial polymerization is non-stationary. The five expanding-window R^2^ values are 0.878, 0.932, 0.960, 0.653, and 0.952 (mean 0.875 ± 0.115; median 0.932). See Table S5. Four windows lie between 0.878 and 0.960, whereas one falls to 0.653. The 0.279 difference between the median and the lowest window is too large to dismiss as trivial numerical variability. It is consistent with a period in which the joint process-quality distribution changed. Formally, this corresponds to the possibility that P_t(X,Y)_ is not invariant with time. The difficult window is therefore retained as evidence of concept drift rather than removed as an outlier. This result provides a direct operational rationale for laboratory reconciliation: periodic ASTM measurements are not merely labels for model development but observations that can re anchor the inferred quality state when the process enters a new regime. Domain-resolved performance further shows why the overall R^2^ = 0.902 must not be interpreted as uniform accuracy across all PP families. Family H contributes 50 of the 65 future observations and achieves R^2^ = 0.930, RMSE = 0.304 wt.%, and MAE = 0.188 wt.%. Only 7.0% of its future variance remains unexplained. The RMSE/MAE ratio is approximately 1.62, indicating that a limited number of larger errors contribute disproportionately to the squared-error metric even though the typical absolute error remains low. See Table 8. These residual deviations may reflect unobserved process variables, residence-time mismatch between DCS conditions and sampled polymer, analytical uncertainty, intrafamily recipe changes, or transition dynamics; they should not automatically be labeled as measurement noise.

### 3.7. Physicochemical and Mathematical Interpretation of the Validated Framework

Family C requires a different statistical interpretation. Its historical XS distribution is narrow (1.408 ± 0.234 wt.%), so the denominator of R^2^, = $SST=\sum_{i}{\left(y_{1}-\bar{y}\right)}^{2}$, is intrinsically small. Consequently, modest absolute prediction errors can produce R^2^ ≤ 0 even when the error is industrially limited. In the final C holdout (*n* = 10), R^2^ = −0.064, RMSE = 0.462 wt.%, and MAE = 0.275 wt.%. For such a low-variance domain, MAE/RMSE and future specification-band classification are more meaningful deployment criteria than maximizing R^2^. Family R presents the opposite limitation: its broader XS distribution is potentially informative, but only five future observations are available. The resulting R^2^ = 0.350, RMSE = 1.992 wt.%, and MAE = 1.538 wt.% are therefore insufficient to estimate prospective performance with acceptable precision. The scientifically supported conclusion is not that R is intrinsically unpredictable, but that the available future sample size is too small to support a deployment claim. Taken together, the results support an information state representation of the problem. Let H_t−1_ denote all certified analytical information available before prediction time and x_t_ the contemporaneous reactor vector. The soft sensor estimates $\hat{x}s_{t}=f(H_{t-1},x_{t})$. Conceptually, this can be viewed as a persistent state contribution g(H_t−1_) plus a dynamic correction h (x_t_|H_t−1_) and unresolved error epsilon t. This is an information decomposition rather than a claim of strict physical additivity. It captures the principal empirical finding: industrial XS is neither a purely instantaneous reactor response nor merely a fixed grade property. Its predictability arises from the interaction between a slowly evolving quality state and faster process perturbations.

### 3.8. Laboratory-Reconciled State Estimation, Applicability Domain, and Limitations

This formulation changes the industrial role of ASTM D5492. The laboratory method remains the certification reference, while the model interpolates the evolving quality state between certified observations. When a new ASTM result becomes available, it updates only future historical descriptors; it never enters the prediction of the observation to which it belongs. The architecture therefore behaves as a sequential state estimator with periodic measurement reconciliation. Such a design is especially appropriate in the presence of the temporal instability identified above, as it provides an explicit mechanism to detect and correct model drift. The applicability domain must nevertheless be stated conservatively. The evidence currently supports strong prospective performance for Family H and useful overall portfolio prediction, but not equivalent deployment claims for C and R. The model should also not be extrapolated to catalyst systems, donor chemistries, reactor configurations, or operating envelopes absent from the development data. Future falsification should focus on prospectively collected campaigns with prespecified sampling intervals, sufficient representation of C and R, explicit grade-transition blocks, and comparison of prediction errors with ASTM D5492 analytical repeatability. Such experiments would distinguish model error from reference-method uncertainty and test whether the historical-plus-dynamic decomposition generalizes beyond the present industrial period.

## 4. Conclusions

This study demonstrates that industrial prediction of the polypropylene xylene-soluble fraction should be formulated as a temporally structured state-estimation problem rather than as a conventional, randomly partitioned tabular regression task. Reanalysis of the original production database showed that apparent predictive performance can be affected by product architecture, historical quality information, temporally neighboring observations, and descriptors that may implicitly encode grade specifications. A leakage-controlled chronological framework was therefore developed in which information available at prediction time was explicitly separated into current reactor-state variables and previously available analytical information. Product code, melt-flow index, contemporaneous laboratory ethylene content, and other downstream quality descriptors were excluded from the primary predictive formulation. The information-decomposition analysis showed that current process measurements alone yielded a temporal-test R^2^ of 0.666, whereas historical analytical/recipe information yielded R^2^ = 0.843. Their integration increased R^2^ to 0.902, with RMSE = 0.640 wt.% and MAE = 0.306 wt.%. Importantly, this corresponds to removal of approximately 37.6% of the variance that remained unexplained by the historical-state model, together with 21.2% and 26.4% reductions in RMSE and MAE, respectively. The contemporaneous reactor state therefore contributes measurable information beyond the persistence of previous laboratory results. Temporal validation further demonstrated non-stationarity (R^2^ = 0.878, 0.932, 0.960, 0.653, and 0.952; mean 0.875 ± 0.115). Family H provided the strongest prospective evidence (*n* = 50, R2 = 0.930, RMSE = 0.304 wt.%, MAE = 0.188 wt.%), whereas the narrow response range of C and the small future sample for R preclude equivalent claims. The lower-performing temporal window and the family-dependent results define the practical applicability limits of the present model rather than being treated as exceptions to it.

From a physicochemical perspective, the revised analysis supports a hierarchical view in which catalyst/donor chemistry and polymer architecture establish the quality domain, historical analytical measurements describe persistence of the recent state, and contemporaneous reactor variables provide additional information associated with deviations from that state. Model-derived associations are consistent with established roles of stereoselectivity control, comonomer incorporation, hydrogen-mediated chain transfer, and reactor operating conditions. Still, they are not interpreted as proof of causal mechanisms. The resulting architecture is best understood as a laboratory reconciled industrial soft sensor. ASTM D5492 remains the certification reference, while the estimator provides intermediate XS predictions between laboratory measurements and is updated only as new certified information becomes available. More broadly, the study shows that trustworthy machine learning applications in multigrade polymer manufacturing require explicit control of information availability, temporal dependence, algebraic redundancy, operating-domain heterogeneity, and the distinction between predictive association and physicochemical causality.

## Figures and Tables

**Table 1:** Industrial variables included in the predictive analysis of the xylene-soluble fraction (XS). ASTM, American Society for Testing and Materials; H_2_, hydrogen; C_2_, ethylene; C_3_, propylene; C_2_H_4_, ethylene; C_3_H_6_, propylene; TEAL, triethylaluminum; Ti, titanium; SCA, selectivity control agent; MF, melt flow; Et, DA, C2PP, and C3PP, process variables as defined in the plant database.

Information Class	Representative Information	Availability	Predictors Included	Primary Use	Rationale
Current reactor state	Gas composition, feed rates, temperature, pressure, residence time, catalyst/SCA	At time *t*	Productivity; H_2_/C_3_; TEAL/Ti; C_2_/C_3_; TEAL/SCA; TEAL Flow; SCA Feed; H_2_ Feed; [C_3_H_6_]; [C_2_H_4_]; [H_2_]; Rate	Yes	Online process information available at the prediction time
Historical analytical state	Previous ASTM XS and lagged/rolling historical descriptors	Before time *t*	Most recently available ASTM XS; lagged XS descriptors; rolling historical XS descriptors; historical recipe information available before *t*	Reconciled model	Provides the previously known analytical/recipe state without using the current XS result
Downstream quality	MF, contemporaneous Et, current XS	After/with laboratory result	MF; contemporaneous Et; current XS; DA; C_2_PP; C_3_PP	No	Unavailable for genuine prediction of XS at time *t* and therefore excluded from the primary model
Product identity	Commercial product code/nominal specification	Known recipe	Commercial product code; nominal product specification	No	May directly or indirectly encode grade-specific XS targets and create information leakage

**Table 2:** Chronological partition used for leakage-controlled model development.

Item	Value
Original records	425
Curated records	425
Development/training	296 (70%)
Temporal validation	63 (15%)
Final temporal test	65 (15%)
Final model	Extra Trees
Validation principle	Chronological/out-of-time
Primary metrics	R^2^, RMSE, MAE
Reference method	ASTM D5492

**Table 3:** Descriptive statistics recalculated from the 425-record curated industrial dataset. MF, Et, DA, C_2_PP, and C_3_PP are reported descriptively but are not used as downstream quality predictors in the primary online model.

Variable	n	Mean	SD	Median	Min	Max	CV (%)	Skewness	Kurtosis
XS	425	3.155	2.085	2.591	0.979	11.780	66.08	1.898	4.267
Productivity	425	25.953	12.853	20.545	6.821	92.637	49.52	1.650	2.964
H_2_/C_3_	425	0.011	0.018	0.004	0.000	0.142	165.27	3.622	16.296
TEAL/Ti	425	46.227	3.525	45.010	43.999	75.152	7.62	3.416	14.592
C_2_/C_3_	74	0.009	0.017	0.003	0.001	0.138	189.27	6.074	43.417
TEAL/SCA	425	2.963	0.676	2.837	1.460	4.572	22.81	0.166	−0.677
TEAL Flow	425	3.804	1.667	3.610	0.910	8.403	43.82	0.330	−0.851
SCA Feed	425	2.949	1.293	2.770	0.803	7.061	43.84	0.683	−0.059
H_2_ Feed	425	2.402	2.331	1.672	0.056	26.507	97.06	4.681	33.241
[C_3_H_6_]	425	84.801	4.432	85.005	39.412	95.528	5.23	−3.163	26.277
[C_2_H_4_]	425	0.101	0.307	0.005	0.000	1.735	305.14	3.867	14.302
[H_2_]	425	0.908	1.476	0.305	0.024	10.899	162.52	3.456	14.308
Rate	425	26.843	6.016	27.303	6.796	38.013	22.41	−0.347	−0.807
MF	425	4.748	8.012	3.187	0.195	61.820	168.74	5.478	32.249
Et	406	1.090	2.642	0.000	0.000	12.500	242.44	2.956	8.194
DA	425	14.397	10.174	20.800	1.185	23.200	70.67	−0.497	−1.709
C_2_PP	406	0.043	0.215	0.002	0.000	3.878	502.12	14.436	250.050
C_3_PP	406	27.771	1.765	27.914	11.694	31.960	6.35	−2.578	17.524

**Table 4:** Descriptive statistics of XS according to the polypropylene family.

Family	n	Mean (wt.%)	SD	Median	Maximum
C	110	1.408	0.234	1.414	2.748
H	278	3.309	1.577	2.864	11.780
R	37	7.195	2.338	7.472	11.120

**Table 5:** Statistical comparison of XS among polypropylene families.

Comparison	*p*-Value	Conclusion
One-way ANOVA	5.59 × 10^−67^	Significant differences among families
Kruskal–Wallis	7.91 × 10^−51^	Significant differences among families

**Table 6:** Tukey post hoc comparison of XS between polypropylene families.

Comparison	Mean Difference	Adjusted *p*-Value	Significant
C vs. H	1.9009	<0.001	Yes
C vs. R	5.7868	<0.001	Yes
H vs. R	3.8859	<0.001	Yes

**Table 7:** Temporal decomposition of information contributing to industrial XS prediction.

Predictive Information	Model	*n* Test	R^2^	RMSE (wt.%)	MAE (wt.%)
Current reactor state	Extra Trees	65	0.666	1.184	0.533
Historical analytical/recipe state	Extra Trees	65	0.843	0.812	0.416
Historical state + current reactor state	Extra Trees	65	0.902	0.640	0.306

**Table 8:** Domain-resolved temporal performance of the final laboratory-reconciled XS soft sensor.

Family	*n* Test	R^2^	RMSE (wt.%)	MAE (wt.%)	Interpretation
H	50	0.930	0.304	0.188	Strongest validated domain
C	10	−0.064	0.462	0.275	Low-variance domain
R	5	0.350	1.992	1.538	Insufficient future observations
Overall	65	0.902	0.640	0.306	Temporal holdout; domain-dependent

## Data Availability

The original contributions presented in this study are included in the article. Further inquiries can be directed to the corresponding author. The industrial dataset contains proprietary production information and cannot be publicly released in full. Aggregated data, methodological details, and non-confidential information required to reproduce the reported analysis may be made available by the corresponding author upon reasonable request, subject to industrial confidentiality restrictions.

## Supplementary Materials

The following supporting information can be downloaded at https://www.mdpi.com/article/10.3390/polym18192401/s1. Table S1: Confidentiality-preserving data-quality and record-curation audit; Table S2: Predictor eligibility by information class and prediction-time availability; Table S3: Missing-data, preprocessing, and leakage-control protocol; Table S4: Final model and temporal-validation protocol; Table S5: Expanding-window temporal-validation performance.
