# Supplementary material for: Temporally Validated and Laboratory-Reconciled Soft Sensing of Xylene-Soluble Fraction in Industrial Polypropylene Manufacturing
Source: Polymers (Basel). 2026 Oct 1;18(19):2401. doi: 10.3390/polym18192401 (PMC13667117; doi:10.3390/polym18192401)
Supplement: Supplementary file 1 [file polymers-18-02401-s001.zip › polymers-4450502-supplementary.pdf]

Supplementary material

This section contains the supplementary material for the study

Table S1. Confidentiality-preserving data-quality and record-curation audit.

Audit item	Initial status	Final treatment	Scientific rationale
Industrial observations	425 records	424 retained	One record failed the predefined physical-consistency audit.
Physically invalid record	1 record	Excluded	A nonphysical combination of feed-state information was identified; exact proprietary values are not disclosed.
Production chronology	Available	Preserved	Required for leakage-resistant temporal validation.
Product identifiers	Available	Excluded from primary model	Could encode grade-specific XS specification.
Current/future XS	Laboratory response	Never used as current predictor	Direct target-leakage prevention.
Downstream QC variables	Available for some records	Excluded from primary online model	Unavailable at the intended prediction time.
Polymer family	Known architecture class	Used for domain analysis/reporting	Prevents a global performance metric from masking family-dependent generalization.

Table S2. Predictor eligibility by information class and prediction-time availability.

Information class	Examples (generic)	Availability	Primary-model status	Reason
Current reactor state	Gas composition; reactor conditions; process-feed descriptors	At prediction time t	Eligible	Represents contemporaneous process state.
Catalyst/donor state	Catalyst and selectivity-control information known before/during production	Before/at t	Eligible where non-confidential and operationally available	Represents recipe/catalytic context without disclosing proprietary formulation.
Historical analytical state	Previously certified ASTM D5492 results and causal lag/rolling summaries	Strictly before t	Eligible	Represents previously known quality state without future leakage.

Product identity	Commercial product/grade code	Known recipe	Excluded	May act as a surrogate for the nominal XS target.
Downstream quality	MF, contemporaneous laboratory Et, current XS and related QC results	After/with laboratory result	Excluded	Not available for genuine online prediction.
Derived descriptors	Ratios or summaries computed from other measurements	At/before t	Audited case-by-case	Avoid duplicate or algebraically dependent information.

Table S3. Missing-data, preprocessing, and leakage-control protocol.

Step	Operation	Implementation	Data allowed	Leakage-control rationale
	Chronological ordering	Industrial records were ordered according to production sequence before any model development or preprocessing.	All records; ordering information only	Preserves temporal dependence and prevents random mixing of past and future observations.
2	Development set	The first 296 chronologically ordered observations were assigned to model development.	Records 1–296	Used to fit preprocessing operations, candidate models, and model parameters.
3	Temporal validation	The subsequent 63 observations were reserved for chronological model validation.	Records 297–359	Used for model and hyperparameter selection without access to the final temporal test set.
4	Final temporal test	The final 65 observations were retained as an independent future holdout.	Records 360–424	One-time out-of-time evaluation after the modeling workflow was frozen.
5	Scenario I: current reactor state	Productivity; H_2/C_3; TEAL/Ti; C_2/C_3; TEAL/SCA; TEAL Flow; SCA Feed; H_2 Feed; $[C_3H_6]$; $[C_2H_4]$; $[H_2]$; Rate.	Measurements available at time t	Contains no current or future laboratory XS information.
6	Scenario II: historical	Most recently available certified ASTM D5492 XS result (XS\(_{lag1}\));	Information strictly	Prevents contemporaneous or future XS from entering the historical predictor set.

	analytical/recipe state	additional causal lagged XS descriptors; rolling summaries of preceding certified XS measurements; historical recipe information available before t.	preceding time t	
7	Scenario III: laboratory-reconciled state	Complete predictor set from Scenario I + complete predictor set from Scenario II.	Information available at or before time t	Quantifies the incremental contribution of contemporaneous reactor information beyond the historical analytical state.
8	Historical XS lag	XS\(_{lag1}\) was defined as the most recent certified ASTM D5492 XS measurement available before the target observation.	Previous certified XS only	The target XS and future XS values were never used.
9	Rolling historical XS features	Rolling descriptors were calculated exclusively from certified XS observations preceding the target observation. The exact window size(s) and statistics used must be reported here from the final modeling code.	Previous certified XS only	No centered or forward-looking windows were permitted.
10	Historical production context	Historical descriptors were restricted to the predefined production context used in the final feature-generation procedure. The exact grouping rule must be stated here (e.g., same family, recipe, or production campaign, according to the actual code).	Previously known context information only	Prevents retrospective assignment using information unavailable at prediction time.

Table S4. Final model and temporal-validation protocol.

Item	Final specification	Comment
Curated observations	424	Chronological industrial records.
Development/training	296 (70%)	Used to fit preprocessing and models.
Temporal validation	63 (15%)	Used for model/hyperparameter selection.
Final temporal test	65 (15%)	Untouched until workflow freeze.
Final algorithm	Extra Trees	Selected under the chronological validation framework.
n_estimators	300	Final configuration.
max_features	0.5	Final configuration.
min_samples_leaf	2	Final configuration.
max_depth	None	Final configuration.
random_state	42	Reproducibility.
Primary metrics	R^2, RMSE, MAE	All calculated from the same prediction vector.

Table S5. Expanding-window temporal-validation performance.

Fold	Training chronological range	n train	Validation chronological range	n validation	R^2
1	Records 1–296	296	Records 297–309	13	0.878
2	Records 1–309	309	Records 310–322	13	0.932
3	Records 1–322	322	Records 323–335	13	0.960
4	Records 1–335	335	Records 336–347	12	0.653
5	Records 1–347	347	Records 348–359	12	0.95

The first 296 chronologically ordered observations constituted the initial development set. The subsequent 63 observations were divided into five consecutive temporal validation blocks of 12–13 observations. After each validation step, the corresponding block was incorporated into the training history, resulting in a progressively expanding training window. No observation occurring after a given validation block was used for training, preprocessing, feature construction, or model selection for that fold. The final 65 observations (records 360–424) constituted an independent temporal holdout and were evaluated only after the modeling workflow had been frozen.
